# Nursing participation in health technology assessment: a pending issue?

**DOI:** 10.1590/1518-8345.0000.4245

**Published:** 2024-11-04

**Authors:** Juan Ramón Lacalle-Remigio, Soledad Benot-López

**Affiliations:** ^1^ Universidad de Sevilla, Departamento de Medicina Preventiva y Salud Pública, Sevilla, Spain.; ^2^ Servicio Andaluz de Salud, Comisiones de Evaluación de Tecnologías Sanitarias, Sevilla, Spain.

**Figure:**
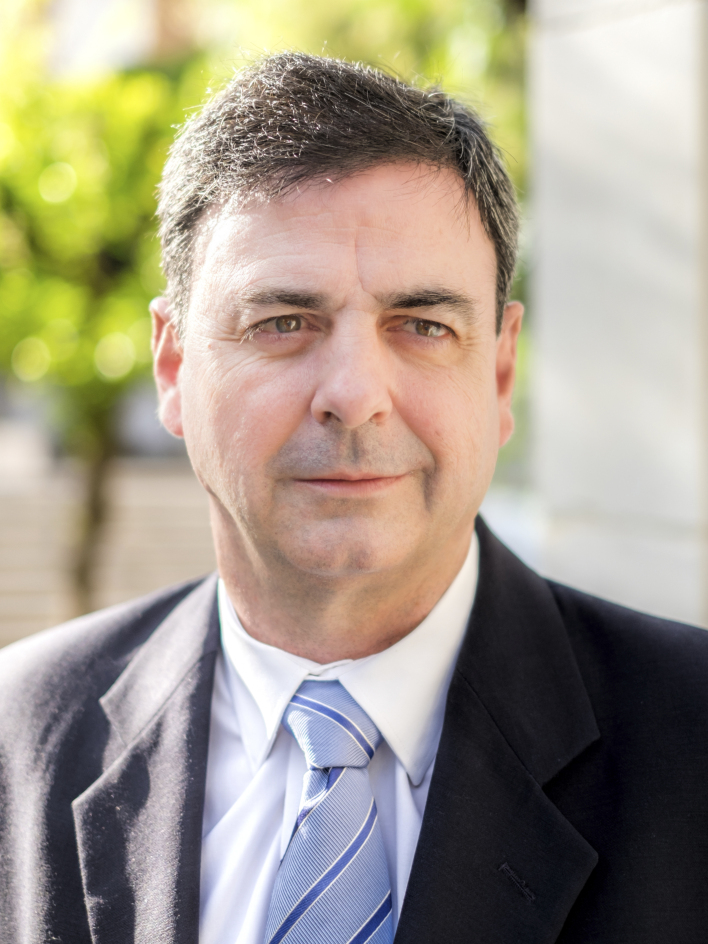

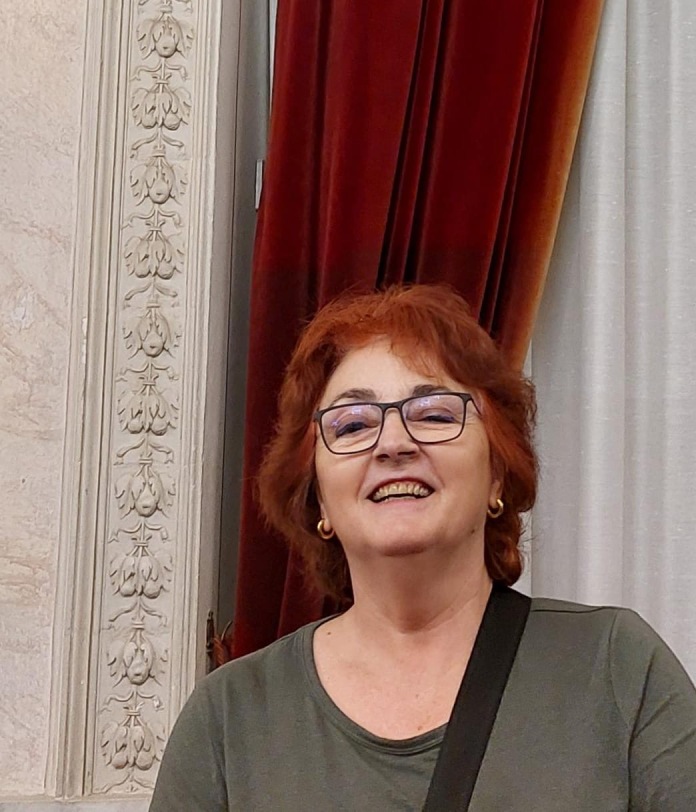

The popular image of a nurse caring for her patients includes a syringe and a thermometer. These two instruments have recently been joined by other electronic instruments and digital resources. For many people, these are examples of health technologies (HT). In reality, however, this term has a much broader meaning. It covers not only medical devices, but also medicines, medical and surgical procedures, procedures for the prevention, diagnosis and treatment of health problems, as well as formulas for the organization and management of healthcare.

 On the other hand, when we talk about health technology assessment (HTA) we refer to a multidisciplinary research process to synthesize the available scientific information. This information concerns the medical, social, economic and ethical consequences of using a health technology. The synthesis must be carried out in a systematic, transparent, unbiased and robust manner ^(^ 1 ^)^ . 

 As a discipline, HTA is relatively young. The first works were published around 1970 ^(^ 2 ^)^ . These articles identified that a large part of the procedures used in healthcare systems had not previously demonstrated their effectiveness. Gradually, many health systems have created organizations to conduct HTA studies, to help them decide on the incorporation of new healthcare procedures. These organizations have been integrated into networks, such as the Latin American one (RedETSA) ^(^ 3 ^)^ or the European one (EunetHTA) ^(^ 4 ^)^ . By working in these networks, participants can harmonize reporting and improve HTA procedures. In addition, patients have recently started to participate in these assessments, providing important added value to their findings. 

 The digitalization of nursing care requires these evaluations, but it is not the only one ^(^ **5** ^)^ . Prevention and treatment of wounds, management of subcutaneous access and reservoirs, catheterization, care of ulcers, of diabetic foot, prevention of pressure injuries or palliative care are some examples in which nursing should participate in the assessment of technologies. To do this, it is necessary for these professionals to become aware of their role and join the teams that carry out this type of research. 

How is HTA research conducted? The data used is obtained from previous studies. For this reason, databases are essential for documentary research. In the field of Nursing, we have specific bibliographic sources (CINAHL, Cuiden, Enfispo, Cuidatge, JBI or EBO Database of the Joanna Briggs Collaboration), research units in health services and nursing care, and NANDA/NOC/NIC nursing diagnosis codes. These resources have undoubtedly helped nursing professionals to conduct research in different fields: primary care, hospital care, teaching institutions and in the different areas of health management.

But this has not happened with HTA research. There are few HTA studies on nursing care. What could be the reasons for this situation? We believe that one of the reasons is that many professionals mistakenly understand that healthcare technologies are only equipment, and for hospital use. As we have indicated before, there are areas of care and organization that can also be assessed. There may be other reasons, such as the limited number of clinical trials evaluating these technologies in nursing. Therefore, there is a lack of scientifically robust evidence.

This means that nursing practice is not sufficiently connected to evidence-based decision-making that affects Nursing professionals. We need more research to evaluate the impact of health technologies on nursing care. HTA is a multidisciplinary field of work, to which professionals with different profiles have been incorporated, including nursing professionals. If they do so, they will provide invaluable insights into the incorporation of new technologies and procedures that improve nursing care and patient outcomes.
