# Supplementary material for: Nursing participation in health technology assessment: a pending issue?
Source: Rev Lat Am Enfermagem. 2024 Nov 4;32:e4245. doi: 10.1590/1518-8345.0000.4245 (PMC11537263; doi:10.1590/1518-8345.0000.4245)
Supplement: Translation

# Translation: Participación de la enfermería en la evaluación de tecnologías sanitarias: ¿una asignatura pendiente?

**DOI:** 10.1590/1518-8345.0000.4244

**Published:** 2024-10-17

**Authors:** Juan Ramón Lacalle-Remigio, Soledad Benot-López

**Affiliations:** ^1^ Universidad de Sevilla, Departamento de Medicina Preventiva y Salud Pública, Sevilla, España.; ^2^ Servicio Andaluz de Salud, Comisiones de Evaluación de Tecnologías Sanitarias, Sevilla, España.

La imagen popular de la enfermera que cuida a sus pacientes incluye la jeringuilla y el termómetro. A estos dos instrumentos se le han unido recientemente otros instrumentos electrónicos y recursos digitales. Para muchas personas, estos son ejemplos de tecnologías sanitarias (TS). Pero en realidad, este término tiene un significado mucho más amplio. Abarca no sólo a los dispositivos médicos, sino que también a los medicamentos, los procedimientos médicos y quirúrgicos, los de prevención, diagnóstico y tratamiento de problemas de salud, así como las fórmulas de organización y gestión sanitaria.

 Por otro lado, cuando hablamos de evaluación de tecnologías sanitarias (ETS) nos referimos a un proceso de investigación multidisciplinar, para sintetizar la información científica disponible. Esta información se refiere a las consecuencias médicas, sociales, económicas y éticas del uso de una tecnología sanitaria. La síntesis se debe realizar de una manera sistemática, transparente, no sesgada y robusta ^(^ 1 ^)^ . 

 Como disciplina, la ETS es relativamente joven. Los primeros trabajos se publicaron alrededor de 1970 ^(^ 2 ^)^ . Estos artículos identificaron que una gran parte de los procedimientos usados en los sistemas sanitarios no habían demostrado previamente su eficacia. Paulatinamente, muchos sistemas sanitarios han creado organismos para hacer estudios de ETS, para ayudarles a decidir sobre la incorporación de nuevos procedimientos asistenciales. Estos organismos se han integrado en redes, como la latinoamericana (RedETSA) ^(^ 3 ^)^ o la europea (EunetHTA) ^(^ 4 ^)^ . Trabajando en estas redes, los participantes pueden armonizar los informes y mejorar los procedimientos de ETS. Además, recientemente los pacientes han empezado a participar en estas evaluaciones, proporcionando un importante valor añadido a sus conclusiones. 

 La digitalización de los cuidados enfermeros necesita de estas evaluaciones, pero no es la única ^(^ 5 ^)^ . La prevención y tratamiento de heridas, el manejo de accesos y reservorios subcutáneos, la canalización de vías, el cuidado de las úlceras, del pie diabético, la prevención de lesiones por presión o los cuidados paliativos, son algunos ejemplos donde la enfermería deberían participar en la evaluación de tecnologías. Para ello, es necesario que estos profesionales tomen conciencia de su papel y se incorporen a los equipos que llevan a cabo este tipo de investigación. 

¿Cómo se investiga en ETS? Los datos usados se obtienen de estudios previos. Por ese motivo, las bases de datos son fundamentales para la búsqueda documental. En el campo de la Enfermería disponemos de fuentes bibliográficas específicas (CINAHL, Cuiden, Enfispo, Cuidatge, JBI o EBO Database de la Joanna Briggs Collaboration), unidades de investigación en servicios de salud y cuidados de enfermería, y códigos de diagnósticos enfermeros NANDA/NOC/NIC. Sin duda, estos recursos han contribuido para que los profesionales de la enfermería investiguen en distintos ámbitos: atención primaria, asistencia hospitalaria, instituciones docentes y en las diferentes áreas de gestión sanitaria.

Pero esto no ha ocurrido con la investigación en ETS. Los estudios de ETS sobre cuidados de enfermería son escasos en número. ¿Cuáles pueden ser las razones que expliquen esta situación? Creemos que uno de los motivos es que muchos profesionales entienden equivocadamente que las tecnologías sanitarias son únicamente equipamientos, y de uso hospitalario. Como hemos indicado antes, hay ámbitos de los cuidados y de la organización que también se pueden evaluar. Pueden existir otras razones, como el número reducido de ensayos clínicos que evalúan estas tecnologías en enfermería. Por ello, hay un déficit de evidencias científicamente robustas.

Ello hace que la práctica de la enfermería no esté suficientemente conectada con la toma de decisiones basadas en la evidencia que afectan a los profesionales de la Enfermería. Necesitamos más investigación para evaluar el impacto de las tecnologías sanitarias en los cuidados enfermeros. La ETS es un campo de trabajo multidisciplinar, al que se han ido incorporando profesionales con distintos perfiles, y caben los profesionales enfermeros. Si lo hacen, aportarán visiones muy valiosas para la incorporación de nuevas tecnologías y procedimientos que mejoren los cuidados enfermeros y los resultados de los pacientes.

# Translation: Participação da enfermagem na avaliação de tecnologias em saúde: um assunto pendente?

**DOI:** 10.1590/1518-8345.0000.4246

**Published:** 2024-10-17

**Authors:** Juan Ramón Lacalle-Remigio, Soledad Benot-López

**Affiliations:** ^1^ Universidad de Sevilla, Departamento de Medicina Preventiva y Salud Pública, Sevilla, Espanha.; ^2^ Servicio Andaluz de Salud, Comisiones de Evaluación de Tecnologías Sanitarias, Sevilla, Espanha.

A imagem popular da enfermeira que cuida de seus pacientes inclui a seringa e o termômetro. A estes dois instrumentos juntaram-se recentemente outros instrumentos eletrônicos e recursos digitais. Para muitas pessoas, estes são exemplos de tecnologias de saúde (TS). Porém, na realidade, este termo tem um significado muito mais amplo. Abrange não apenas dispositivos médicos, mas também medicamentos, procedimentos médicos e cirúrgicos, prevenção, diagnóstico e tratamento de problemas de saúde, bem como fórmulas de organização e gestão da saúde.

 Por outro lado, quando falamos em avaliação de tecnologias em saúde (ATS) referimo-nos a um processo de pesquisa multidisciplinar, para sintetizar a informação científica disponível. Essa informação refere-se às consequências médicas, sociais, econômicas e éticas da utilização de uma tecnologia em saúde. A síntese deve ser realizada de forma sistemática, transparente, imparcial e robusta ^(^ 1 ^)^ . 

 Como disciplina, a ATS é relativamente jovem. Os primeiros trabalhos foram publicados por volta de 1970 ^(^ 2 ^)^ . Esses artigos identificaram que grande parte dos procedimentos utilizados nos sistemas de saúde não havia demonstrado anteriormente sua eficácia. Gradualmente, muitos sistemas de saúde criaram organizações para realizar estudos sobre ATS, para ajudá-los a decidir sobre a incorporação de novos procedimentos de cuidado. Essas organizações foram integradas em redes, como a latino-americana (RedETSA) ^(^ 3 ^)^ ou a europeia ( *EunetHTA* ) ^(^ 4 ^)^ . Ao trabalhar nessas redes, os participantes podem harmonizar os relatórios e melhorar os procedimentos de ATS. Além disso, os pacientes começaram recentemente a participar nessas avaliações, proporcionando um importante valor agregado às suas conclusões. 

 A digitalização dos cuidados de enfermagem precisa dessas avaliações, mas não é a única ^(^ 5 ^)^ . A prevenção e o tratamento de feridas, a gestão de acessos e reservatórios subcutâneos, o cateterismo, o cuidado de úlceras, do pé diabético, a prevenção de lesões por pressão ou cuidados paliativos, são alguns exemplos em que a enfermagem deve participar na avaliação de tecnologias. Para isso, é necessário que esses profissionais tenham consciência do seu papel e integrem as equipes que realizam esse tipo de pesquisa. 

 Como a pesquisa em ATS é realizada? Os dados usados são obtidos de estudos anteriores. Por esse motivo, bancos de dados são essenciais para a pesquisa documental. Na área da Enfermagem, dispomos de fontes bibliográficas específicas (CINAHL, Cuiden, Enfispo, Cuidatge, JBI ou EBO *Database* da *Joanna Briggs Collaboration* ), unidades de pesquisa em serviços de saúde e cuidados de enfermagem, e códigos de diagnósticos de enfermagem NANDA/NOC/NIC. Sem dúvida, esses recursos têm contribuído para que os profissionais de enfermagem pesquisem em diferentes campos: atenção básica, atenção hospitalar, instituições de ensino e em diferentes áreas da gestão em saúde. 

Mas isso não aconteceu com a pesquisa em ATS. Os estudos em ATS sobre cuidados de enfermagem são poucos. Quais poderiam ser as razões que explicam essa situação? Acreditamos que um dos motivos é que muitos profissionais entendem erroneamente que as tecnologias em saúde são apenas equipamentos e de uso hospitalar. Como já indicamos, existem áreas de cuidado e organização que também podem ser avaliadas. Pode haver outros motivos, como o pequeno número de ensaios clínicos que avaliam essas tecnologias na enfermagem. Portanto, faltam evidências cientificamente robustas.

Isso significa que a prática de enfermagem não está suficientemente ligada à tomada de decisão baseada em evidências que afeta os profissionais de Enfermagem. Precisamos de mais pesquisas para avaliar o impacto das tecnologias em saúde nos cuidados de enfermagem. A ATS é um campo de trabalho multidisciplinar, ao qual vêm se agregando profissionais de diferentes perfis, inclusive profissionais de enfermagem. Se o fizerem, fornecerão visões muito valiosas para a incorporação de novas tecnologias e procedimentos que melhorem os cuidados de enfermagem e os resultados dos pacientes.
